# Investigation of the Near-Tip Stress Field of a Notch Terminating at a Bi-Material Interface

**DOI:** 10.3390/ma14164466

**Published:** 2021-08-09

**Authors:** Grzegorz Mieczkowski, Dariusz Szpica, Andrzej Borawski, Mohamed M. Awad, Ahmed Elgarayhi, Mohammed Sallah

**Affiliations:** 1 Faculty of Mechanical Engineering, Bialystok University of Technology, 45C Wiejska Str., 15-351 Bialystok, Poland; d.szpica@pb.edu.pl (D.S.); a.borawski@pb.edu.pl (A.B.); 2 Faculty of Engineering, Mansoura University, Mansoura 35516, Egypt; m_m_awad@mans.edu.eg; 3 Faculty of Science, Mansoura University, Mansoura 35516, Egypt; elgarayhi@mans.edu.eg (A.E.); msallahd@mans.edu.eg (M.S.)

**Keywords:** interface fracture, V-notch in bi-materials, singular stress fields, stress intensity factors

## Abstract

The article deals with the problem of a sharp corner, the tip of which is located on the bi-material interface. The paper presents a qualitative and quantitative description of singular stress fields occurring in the tip area of such a stress concentrator. The qualitative description was obtained by solving the problem of the plane theory of elasticity with appropriately defined boundary conditions. To obtain a quantitative description, it was necessary to determine the values of generalised stress intensity factors (GSIFs). The GSIFs were determined using the developed analytical-numerical method. The calculations were made for various load variants (uniaxial/biaxial tension load, shear load) and notch positions (single/double edge-notched plate, centre-notched plate). Additionally, the impact of notch geometry (height and opening angle) and relative stiffness (Young’s moduli ratio of both components of bi-material) on GSIFs was investigated. It has been noticed that with a decrease in the relative stiffness and an increase in the notch angle or its height, the normalised GSIFs values increased. The obtained results were compared with the data available in the literature and their satisfactory agreement with those presented by other scientists was found.

## 1. Introduction

Ensuring the high durability of the structure with minimum costs is a priority of today’s world economy. The durability of the structure largely depends on the strength of the materials used for its components.

Currently, various types of composites are widely used, which, compared to traditional construction materials, are characterised by greater strength and at the same time lower specific weight. Composites are not without some drawbacks. They often contain various material defects (voids, inclusions) causing the formation of local stress fields with large gradients. This results in the initiation of new cracks or the propagation of the existing ones. In the case where the stress concentrator is located inside only one material phase, the failure criteria commonly used for isotropic materials can be used to predict fracture [1,2,3,4]. In the opposite situation, e.g., when the defect is located or started at the surface of the connection of different elastic materials, it is necessary to develop new, or modify already existing, material fracture criteria. Suitable fracture hypotheses can be obtained using the qualitative and quantitative description of singular stress fields occurring in close proximity to the tip of the defect under consideration.

Many scientists have dealt with the analytical description of local mechanical fields generated by defects located at the interface (e.g., crack [5,6], structural notch [7,8], and rigid inclusion [9,10,11]). In the literature, much less attention has been paid to the problem of a notch whose tip is located at the interface. Such a defect may appear as a result of the development of voids located in one of the phases of the composite [12,13,14]. It is worth noting that a special case of such a sharp corner (notch with a zero-opening angle) is the crack initiated at the interface [15,16,17,18,19,20,21,22,23,24]. Stress singularities occurring in the tip area of such crack, which is perpendicular to the interface were analysed in [15]. GSIFs values, determined for a short crack located in a bi-material with infinite and finite overall dimensions, are presented in [16] and [17,18,19,20,21,22,23], respectively. The problem of local stress concentration, occurring in the vicinity of the crack tip, not perpendicular to the interface, was discussed in [24,25]. As for the corners with a non-zero opening angle, the authors of the papers [12,26] have dealt with this subject. In [26], eigenequation was derived and the influence of material constants on the roots of this equation was investigated. Moreover, the authors, assuming that the notched bi-material is subjected to tensile loads, determined generalised stress intensity factors. An approximate analytical model, based on the theory of multilayer beams, enabling the calculation of GSIF (for mode I loading) is also presented in [12].

There is no insufficient information in the literature on the complete description of the singular stress fields generated by a notch whose tip is at the interface of two elastic bodies. Therefore, the main goal of the work was to obtain such analytical and numerical solutions. The obtained analytical descriptions (eigenequation, formulas for individual components of the stress tensor) along with the proposed method of GSIFs determination are presented in the first part of the presented work. The values of the generalised stress intensity factors, determined for various load variants and geometric and material properties of the composite, are presented in the second part of the article.

## 2. Materials and Methods

### 2.1. Analytical Solutions 

**Figure 1:**
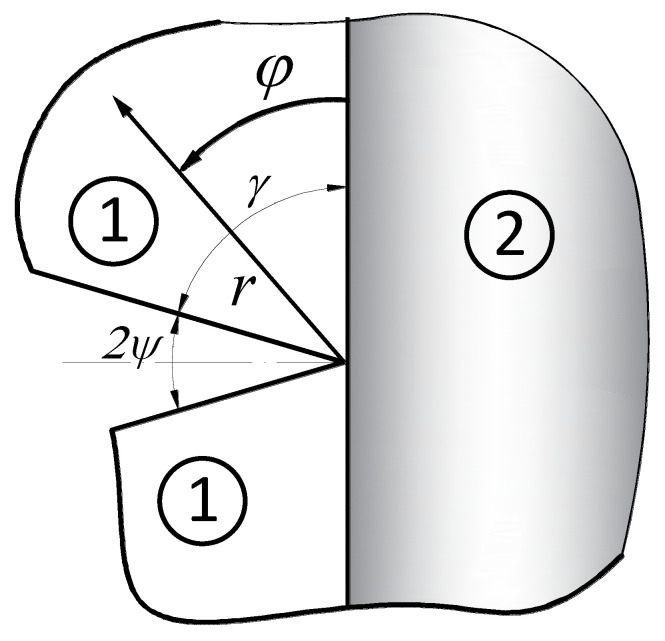
A notch terminating at a bi–material interface.

The analytical description of the stress fields was obtained by solving (with the accuracy of multiplicative constants called GSIFs) a plane problem of two connected elastic half-spaces, the interface of which is weakened by a sharp corner (Figure 1).

Consider a sharp corner (Figure 1) with a polar coordinate system (with coordinates *r* and *φ*) which is located at the notch tip. In such a reference system, there are two independent components of the displacement vector, in the radial (*u_r_*) and angular direction (*u_φ_*). 

The related strain components have the following form:

(1) $$\varepsilon_{r}=\frac{\partial u_{r}}{\partial r},\;\varepsilon_{\varphi }=\frac{1}{r}\frac{\partial u_{\varphi }}{\partial \varphi }+\frac{u_{r}}{r},\;\varepsilon_{r\varphi }=\frac{1}{r}\frac{\partial u_{r}}{\partial \varphi }+\frac{\partial u_{\varphi }}{\partial r}-\frac{u_{\varphi }}{r}.$$

The generalised Hooke’s law can be written as:

(2) $$\sigma_{r}=\Lambda \left(\varepsilon_{r}+\varepsilon_{\varphi }\right)+2\mu \varepsilon_{r},\;\sigma_{\varphi }=\Lambda \left(\varepsilon_{r}+\varepsilon_{\varphi }\right)+2\mu \varepsilon_{\varphi },\;\tau_{r\varphi }=\mu \varepsilon_{r\varphi }\;$$

where *Λ*, *μ* are Lame’s constants and are respectively: $\Lambda =\frac{E\nu }{\left(1+\nu \right)\left(1-2\nu \right)},\mu =\frac{E}{2\left(1+\nu \right)}.$

Navier equations are described by the Formula (3):

(3) $$\frac{\partial \sigma_{r}}{\partial r}+\frac{1}{r}\frac{\partial \tau_{r\varphi }}{\partial \varphi }+\frac{\sigma_{r}-\sigma_{\varphi }}{r}=0,\;\frac{\partial \tau_{r\varphi }}{\partial r}+\frac{1}{r}\frac{\partial \sigma_{\varphi }}{\partial \varphi }+2\frac{\tau_{r\varphi }}{r}=0.\;$$

By using Formulas (1)–(3), equilibrium equations (Lame’s equation) can be obtained:

(4) $$\begin{array}{l} \left(\Lambda +2\mu \right)\frac{\partial }{\partial r}\left(\frac{\partial u_{r}}{\partial r}+\frac{1}{r}\frac{\partial u_{\varphi }}{\partial \varphi }+\frac{u_{r}}{r}\right)-\mu \frac{1}{r}\frac{\partial }{\partial \varphi }\left(\frac{\partial u_{\varphi }}{\partial r}-\frac{1}{r}\frac{\partial u_{r}}{\partial \varphi }+\frac{u_{\varphi }}{r}\right)=0\\ \left(\Lambda +2\mu \right)\frac{1}{r}\frac{\partial }{\partial \varphi }\left(\frac{\partial u_{r}}{\partial r}+\frac{1}{r}\frac{\partial u_{\varphi }}{\partial \varphi }+\frac{u_{r}}{r}\right)+\mu \frac{\partial }{\partial r}\left(\frac{\partial u_{\varphi }}{\partial r}-\frac{1}{r}\frac{\partial u_{r}}{\partial \varphi }+\frac{u_{\varphi }}{r}\right)=0 \end{array}\}.$$

The asymptotic form of the displacement function can be written as [27,28]:

(5) $$u_{r}\left(r,\varphi \right)=r^{\lambda }f\left(\varphi \right),\;u_{\varphi }\left(r,\varphi \right)=r^{\lambda }g\left(\varphi \right),\;$$

where *f*(*φ*) and *g*(*φ*) are angular coordinate functions obtained on the basis of the Airy stress function.

Substituting (5) to Formula (4), a system of differential equations was obtained, from which the general form of the asymptotic solution is determined (6) [11,28]:

(6) $$\begin{array}{l} u_{ri}=r^{\lambda_{j}}\left(A_{i}\;\cos\left(\left(1+\lambda_{j}\right)\varphi \right)+B_{i}\;\sin\left(\left(1+\lambda_{j}\right)\varphi \right)+C_{i}\;\cos\left(\left(1-\lambda_{j}\right)\varphi \right)+D_{i}\;\sin\left(\left(1-\lambda_{j}\right)\varphi \right)\right)\\ u_{\varphi i}=r^{\lambda_{j}}\left(-A_{i}\;\sin\left(\left(1+\lambda_{j}\right)\varphi \right)+B_{i}\;\cos\left(\left(1+\lambda_{j}\right)\varphi \right)-C_{i}\frac{\kappa_{i}+\lambda_{j}}{\kappa_{i}-\lambda_{j}}\;\sin\left(\left(1-\lambda_{j}\right)\varphi \right)+D_{i}\frac{\kappa_{i}+\lambda_{j}}{\kappa_{i}-\lambda_{j}}\;\cos\left(\left(1-\lambda_{j}\right)\varphi \right)\right)\\ \sigma_{ri}=r^{\lambda_{j}-1}\mu_{i}\left(A_{i}2\lambda_{j}\cos\left(\left(1+\lambda_{j}\right)\varphi \right)+B_{i}2\lambda_{j}\sin\left(\left(1+\lambda_{j}\right)\varphi \right)+C_{i}\left(3-\lambda_{j}\right)\frac{2\lambda_{j}}{\kappa_{i}-\lambda_{j}}\cos\left(\left(1-\lambda_{j}\right)\varphi \right)+D_{i}\left(3-\lambda_{j}\right)\frac{2\lambda_{j}}{\kappa_{i}-\lambda_{j}}\sin\left(\left(1-\lambda_{j}\right)\varphi \right)\right)\\ \sigma_{\varphi i}=r^{\lambda_{j}-1}\mu_{i}\left(-A_{i}2\lambda_{j}\cos\left(\left(1+\lambda_{j}\right)\varphi \right)-B_{i}2\lambda_{j}\sin\left(\left(1+\lambda_{j}\right)\varphi \right)+C_{i}\left(1+\lambda_{j}\right)\frac{2\lambda_{j}}{\kappa_{i}-\lambda_{j}}\cos\left(\left(1-\lambda_{j}\right)\varphi \right)+D_{i}\left(1+\lambda_{j}\right)\frac{2\lambda_{j}}{\kappa_{i}-\lambda_{j}}\sin\left(\left(1-\lambda_{j}\right)\varphi \right)\right)\\ \tau_{r\varphi i}=r^{\lambda_{j}-1}\mu_{i}\left(-A_{i}2\lambda_{j}\sin\left(\left(1+\lambda_{j}\right)\varphi \right)+B_{i}^{}2\lambda_{j}\cos\left(\left(1+\lambda_{j}\right)\varphi \right)+C_{i}\left(1-\lambda_{j}\right)\frac{2\lambda_{j}}{\kappa_{i}-\lambda_{j}}\sin\left(\left(1-\lambda_{j}\right)\varphi \right)-D_{i}\left(1-\lambda_{j}\right)\frac{2\lambda_{j}}{\kappa_{i}-\lambda_{j}}\cos\left(\left(1-\lambda_{j}\right)\varphi \right)\right) \end{array}\},\;$$

where: $\mu_{i}=\frac{E_{i}}{2\left(1+\nu_{i}\right)}$—shear modulus $\kappa_{i}=\left(3-\nu_{i}\right)/\left(1+\nu_{i}\right)$—a plane stress, $\kappa_{i}=\left(3-4\nu_{i}\right)$—a plane strain, *ν_i_*—Poisson’s ratio, *i* = 1, 2, *j* = *I* for symmetric problems (Mode I), *j* = *II* for skew-symmetric problems (Mode II).

Particular solutions for the analysed plane problem of notched bi-material were derived by determining the constants *A_i_*, *B_i_*, *C_i_*, *D_i_*_,_ and eigenvalue λ*_j_*. The unknowns sought were obtained on the basis of the following boundary conditions:

1. along the interface, for *φ* = 0 [29];
  $u_{r1}=u_{r2};u_{\varphi 1}=u_{\varphi 2};\sigma_{\varphi 1}=\sigma_{\varphi 2};\tau_{r\varphi 1}=\tau_{r\varphi 2}$,
2. of the upper surface of the V-notch, for *φ* = *γ*;
  $\sigma_{\varphi 1}=\tau_{r\varphi 1}=0$
3. for φ=−π/2
  - symmetry conditions (Mode I)
    $\tau_{r}{}_{\varphi 2}=u_{\varphi 2}=0$
  - skew-symmetry conditions (Mode II)
    $\sigma_{\varphi 2}=u_{r2}=0$

Eigenequations (7-symmetric problem, 8-skew-symmetric problem) from the zero condition of the boundary condition matrix determinant were determined:

(7) $$\begin{array}{l} \beta \lambda_{I}\sin\left[2\gamma \right]+\left(\beta -1+2\alpha \left(-1-\alpha +\beta +\left(\alpha -\beta \right)\lambda_{I}{}^{2}\right)+2\alpha \left(\beta -\alpha \right)\lambda_{I}{}^{2}\cos\left[2\gamma \right]\right)\sin\left[\pi \lambda_{I}\right]+\\ +\alpha \left(1+\alpha -\beta \right)\sin\left[\left(\pi -2\gamma \right)\lambda_{I}\right]+\left(1+\alpha \right)\left(\alpha -\beta \right)\sin\left[\left(\pi +2\gamma \right)\lambda_{I}\right]=0,\; \end{array}$$

(8) $$\begin{array}{l} \beta \lambda_{II}\sin\left[2\gamma \right]+\left(1-\beta +2\alpha \left(1+\alpha -\alpha \lambda_{II}{}^{2}+\beta \left(\lambda_{II}{}^{2}-1\right)\right)+2\alpha \left(\alpha -\beta \right)\lambda_{II}{}^{2}\cos\left[2\gamma \right]\right)\sin\left[\pi \lambda_{II}\right]+\\ +\alpha \left(\beta -\alpha -1\right)\sin\left[\left(\pi -2\gamma \right)\lambda_{II}\right]-\left(1+\alpha \right)\left(\alpha -\beta \right)\sin\left[\left(\pi +2\gamma \right)\lambda_{II}\right]=0,\; \end{array}$$

where: $\alpha =\frac{\mu_{1}/\mu_{2}-1}{\left(1+\kappa_{1}\right)},\beta =\frac{\mu_{1}\left(1+\kappa_{2}\right)}{\mu_{2}\left(1+\kappa_{1}\right)}.$

The roots of the above equations correspond to the eigenvalues λ*_j_*. 

Assuming in Formulas (7) and (8) that α=0,β=1,γ=γ−π/2, the resulting eigenequations are identical to those for the notch problem in isotropic material [27]:

(9) $$\lambda_{I}\sin\left[2\gamma \right]+\sin\left[2\gamma \lambda_{I}\right]=0,\;\lambda_{II}\sin\left[2\gamma \right]-\sin\left[2\gamma \lambda_{I}\right]=0.$$

**Figure 2:**
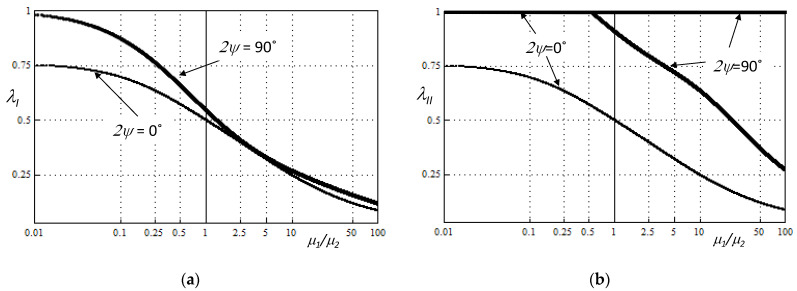
A solution of eigenequations (*ν*_1_ = *ν*_2_ = 0.3, plane stress condition), (**a**) for Mode I (7), (**b**) for Mode II (8).

The roots of Equations (7) and (8) cannot be found analytically. They were determined numerically using the Berents method applied to the proprietary calculation program written in the Wolfram Language. The eigenvalues λ*_j_* obtained in this way, determined for notches with arbitrarily assumed opening angles, are shown graphically in Figure 2.

It was found that the parameters λ*_j_*, regardless of the notch geometry and the mechanical properties of the bi-material, always assume real values. Moreover, the strength of the stress singularity increases with a decrease in the notch angle and an increase in the relative stiffness.

To obtain an analytical description of individual components of the stress tensor, it was necessary to define GSIFs in advance. Since eigenvalues *λ_j_* always assume real values, the generalised stress intensity factors *K_j_* were defined, similar to the work [27], as follows:

(10) $$\begin{array}{l} K_{I}^{}=\underset{r\to 0}{\lim}\sqrt{2\pi }r^{1-\lambda_{I}}\sigma_{\varphi 2}(r,-\pi /2)\\ K_{II}^{}=\underset{r\to 0}{\lim}\sqrt{2\pi }r^{1-\lambda_{II}}\tau_{r\varphi 2}(r,-\pi /2) \end{array}\}.$$

Due to the rather complicated form of the solutions obtained, only the stress formulas in the material in which the potential crack will propagate are presented below (for the notch shown in Figure 1 it is the material marked with the number 2).

(11) $$\begin{array}{l} \sigma_{\varphi 2}=\left(\begin{array}{l} \frac{-K_{I}r^{\lambda_{I}-1}\left(\left(1+\lambda_{I}\right)\cos\left[\frac{1}{2}\left(\lambda_{I}-1\right)\left(\pi +2\varphi \right)\right]F_{I2}+\alpha \mu_{1}\left(\lambda_{I}-\kappa_{2}\right)\sin\left[\frac{\pi \lambda_{I}}{2}+\varphi +\lambda_{I}\varphi \right]F_{I1}\right)}{\sqrt{2\pi }\alpha \mu_{1}\left(\lambda_{I}-\kappa_{2}\right)F_{I0}}+\\ \frac{K_{II}r^{\lambda_{II}-1}\left(\left(1+\lambda_{II}\right)\cos\left[\frac{\pi \lambda_{II}}{2}+\left(\lambda_{II}-1\right)\varphi \right]F_{II2}+\alpha \left(\lambda_{II}-\kappa_{2}\right)\cos\left[\frac{\pi \lambda_{II}}{2}+\varphi +\lambda_{II}\varphi \right]F_{II1}\right)}{\sqrt{2\pi }\alpha \mu_{1}\left(\lambda_{II}-\kappa_{2}\right)F_{II0}} \end{array}\right)\\ \tau_{r\varphi 2}=\left(\begin{array}{l} \frac{K_{I}r^{\lambda_{I}-1}\left(\left(\lambda_{I}-1\right)\cos\left[\frac{\pi \lambda_{I}}{2}+\left(\lambda_{I}-1\right)\varphi \right]F_{I2}+\alpha \mu_{1}\left(\lambda_{I}-\kappa_{2}\right)\cos\left[\frac{\pi \lambda_{I}}{2}+\varphi +\lambda_{I}\varphi \right]F_{I1}\right)}{\sqrt{2\pi }\alpha \mu_{1}\left(\lambda_{I}-\kappa_{2}\right)F_{I0}}+\\ +\frac{K_{II}r^{\lambda_{II}-1}\left(\left(\lambda_{II}-1\right)\cos\left[\frac{1}{2}\left(\lambda_{II}-1\right)\left(\pi +2\varphi \right)\right]F_{II2}+\alpha \left(\lambda_{II}-\kappa_{2}\right)\sin\left[\frac{\pi \lambda_{II}}{2}+\varphi +\lambda_{II}\varphi \right]F_{II1}\right)}{\sqrt{2\pi }\alpha \mu_{1}\left(\lambda_{II}-\kappa_{2}\right)F_{II0}} \end{array}\right)\\ \sigma_{r2}=\left(\begin{array}{l} \frac{K_{I}r^{\lambda_{I}-1}\left(\left(\lambda_{I}-3\right)\cos\left[\frac{1}{2}\left(\lambda_{I}-1\right)\left(\pi +2\varphi \right)\right]F_{I2}+\alpha \mu_{1}\left(\lambda_{I}-\kappa_{2}\right)\sin\left[\frac{\pi \lambda_{I}}{2}+\varphi +\lambda_{I}\varphi \right]F_{I1}\right)}{2\sqrt{2\pi }\alpha \mu_{1}\left(\lambda_{I}-\kappa_{2}\right)F_{I0}}+\\ -\frac{K_{II}r^{\lambda_{II}-1}\left(\left(\lambda_{II}-3\right)\cos\left[\frac{\pi \lambda_{II}}{2}+\left(\lambda_{II}-1\right)\varphi \right]F_{II2}+\alpha \left(\lambda_{II}-\kappa_{2}\right)\cos\left[\frac{\pi \lambda_{II}}{2}+\varphi +\lambda_{II}\varphi \right]F_{II1}\right)}{\sqrt{2\pi }\alpha \mu_{1}\left(\lambda_{II}-\kappa_{2}\right)F_{II0}} \end{array}\right) \end{array}\},\;$$

where:

$$F_{I1}=\left(\begin{array}{l} -\alpha \left(\lambda_{I}-1\right)\lambda_{I}\cos\left[\gamma +\frac{\pi \lambda_{I}}{2}-\gamma \lambda_{I}\right]+\left(1+\alpha -\beta +\alpha \lambda_{I}{}^{2}\right)\cos\left[\gamma -\frac{\pi \lambda_{I}}{2}+\gamma \lambda_{I}\right]+\\ +\left(\beta -2\alpha -1\right)\lambda_{I}\cos\left[\gamma +\frac{\pi \lambda_{I}}{2}+\gamma \lambda_{I}\right]+\left(\alpha -\beta \right)\left(\lambda_{I}-1\right)\cos\left[\gamma -\frac{1}{2}\left(\pi +2\gamma \right)\lambda_{I}\right] \end{array}\right),$$

$$F_{I2}=\left(\alpha -\beta +\alpha \lambda_{I})\mu_{1}+\beta \mu_{2}\right)\left(\cos\left[\gamma +\frac{\pi \lambda_{I}}{2}+\gamma \lambda_{I}\right]-2\alpha \left(\lambda_{I}\sin\left[\gamma \right]\sin\left[\frac{1}{2}\left(\pi -2\gamma \right)\lambda_{I}\right]+\sin\left[\gamma \lambda_{I}\right]\sin\left[\gamma +\frac{\pi \lambda_{I}}{2}\right]\right)\right),$$

$$F_{I0}=\left(\begin{array}{l} 2\cos\left[\gamma \right]\left(-\lambda_{I}\cos\left[\frac{\pi \lambda_{I}}{2}\right]\cos\left[\gamma \lambda_{I}\right]+\left(1-\beta +\lambda_{I}+2\alpha \left(1+\lambda_{I}\right)\right)\sin\left[\frac{\pi \lambda_{I}}{2}\right]\sin\left[\gamma \lambda_{I}\right]\right)+\\ +\sin\left[\gamma \right]\left(\left(1-\beta +4\alpha \lambda_{I}{}^{2}\right)\sin\left[\frac{1}{2}\left(\pi -2\gamma \right)\lambda_{I}\right]+\left(1+\beta +2\lambda_{I}+4\alpha \lambda_{I}-2\beta \lambda_{I}\right)\sin\left[\frac{1}{2}\left(\pi +2\gamma \right)\lambda_{I}\right]\right) \end{array}\right),$$

$$F_{II1}=\mu_{1}\left(\begin{array}{l} \alpha \lambda_{II}\left(1+\lambda_{II}\right)\cos\left[\gamma +\frac{\pi \lambda_{II}}{2}-\gamma \lambda_{II}\right]-\left(1+\alpha -\beta +\alpha \lambda_{II}^{2}\right)\cos\left[\gamma -\frac{\pi \lambda_{II}}{2}+\gamma \lambda_{II}\right]+\\ \left(\beta -1-2\alpha \right)\lambda_{II}\cos\left[\gamma +\frac{\pi \lambda_{II}}{2}+\gamma \lambda_{II}\right]+\left(\alpha -\beta \right)\left(1+\lambda_{II}\right)\cos\left[\gamma -\frac{1}{2}\left(\pi +2\gamma \right)\lambda_{II}\right] \end{array}\right),$$

$$F_{II2}=\left(\left(\alpha -\beta +\alpha \lambda \right)\mu_{1}+\beta \mu_{2}\right)\left(\begin{array}{l} \left(1+\alpha \right)\cos\left[\gamma +\frac{\pi \lambda_{II}}{2}+\gamma \lambda_{II}\right]+\\ +\alpha \lambda_{II}\cos\left[\gamma -\frac{\pi \lambda_{II}}{2}+\gamma \lambda_{II}\right]-\alpha \left(1+\lambda_{II}\right)\cos\left[\gamma +\frac{\pi \lambda_{II}}{2}-\gamma \lambda_{II}\right] \end{array}\right),$$

$$F_{II0}=\left(\begin{array}{l} 2\cos\left[\gamma \right]\left(\lambda_{II}\cos\left[\frac{\pi \lambda_{II}}{2}\right]\cos\left[\gamma \lambda_{II}\right]-\left(\beta -1+2\alpha \left(\lambda_{II}-1\right)+\lambda_{II}\right)\sin\left[\frac{\pi \lambda_{II}}{2}\right]\sin\left[\gamma \lambda_{II}\right]\right)+\\ +\sin\left[\gamma \right]\left(\left(1-\beta +4\alpha \lambda_{II}^{2}\right)\sin\left[\frac{1}{2}\left(\pi -2\gamma \right)\lambda_{II}\right]+\left(1+\beta +2\left(\beta -1-2\alpha \right)\lambda_{II}\right)\sin\left[\frac{1}{2}\left(\pi +2\gamma \right)\lambda_{II}\right]\right) \end{array}\right).$$

For the quantitative description of stresses, it is necessary to determine the values of the *K_j_* coefficients (GSIFs). A method for finding generalised stress intensity factors is discussed in the next section.

### 2.2. The Method for Determining Generalised Stress Intensity Factors K_j_

For the considered problem of a notch with a tip located on the bi-material interface, there are no exact solutions enabling the determination of the value of generalised stress intensity factors. The multiplied constants *K_j_* (GSIFs) used in Formula (11) can be found using approximate methods. In this paper, the analytical and numerical method for determining GSIFs presented in the work [30] was used. It is an asymptotic method, based on the comparison of analytically and numerically obtained stress distributions in the vicinity of the defect’s tip. So, for its application, it was necessary to derive, based on the obtained analytical solutions, approximating functions and to determine the appropriate stresses using numerical methods (finite element method (FEM)). The methodology of obtaining the latter is discussed in the next section. Below, the approach of the applied method of GSIFs determination is discussed and the formulas for approximating functions are derived.

The following functions were used to extrapolate the hoop *σ_φ_*_2_ and tangential *τ_rφ_*_2_ stresses numerically determined at two neighbouring points located at a distance of *r_n_* and *r_n_*_+1_ from the notch tip:

(12) $$\begin{array}{l} \sigma_{\varphi 2\left(r_{n},-\pi /2\right)}=\frac{K_{I(r)}}{\sqrt{2\pi }r^{1-\lambda_{I}}}\left(1+c_{I}r_{n}\right),\sigma_{\varphi 2\left(r_{n}+1,-\pi /2\right)}=\frac{K_{I(r)}}{\sqrt{2\pi }r^{1-\lambda_{I}}}\left(1+c_{I}r_{n+1}\right)\\ \tau_{r\varphi 2\left(r_{n},-\pi /2\right)}=\frac{K_{II(r)}}{\sqrt{2\pi }r^{1-\lambda_{II}}}\left(1+c_{II}r_{n}\right),\tau_{r\varphi 2\left(r_{n}+1,-\pi /2\right)}=\frac{K_{II(r)}}{\sqrt{2\pi }r^{1-\lambda_{II}}}\left(1+c_{II}r_{n+1}\right) \end{array}\}$$

where *C_I_*, *C_II_* are constants that can be eliminated from the equations.

Applying the (10) and (11) to the extrapolating functions (12), the formulas for GSIFs (approximating functions) were obtained in the following form:

(13) $$\begin{array}{l} K_{I(r)}^{}=\frac{\sqrt{2\pi }{\left(r_{n}r_{n+1}\right)}^{1-\lambda_{I}}\left(r_{n}{}^{\lambda_{I}}\sigma_{\varphi 2\left(r_{n}+1,-\pi /2\right)}-r_{n+1}{}^{\lambda_{I}}\sigma_{\varphi 2\left(r_{n},-\pi /2\right)}\right)}{r_{n}-r_{n+1}}\\ K_{II(r)}^{}=\frac{\sqrt{2\pi }{\left(r_{n}r_{n+1}\right)}^{1-\lambda_{II}}\left(r_{n}{}^{\lambda_{II}}\tau_{r\varphi 2\left(r_{n}+1,-\pi /2\right)}-r_{n+1}{}^{\lambda_{II}}\tau_{r\varphi 2\left(r_{n},-\pi /2\right)}\right)}{r_{n}-r_{n+1}} \end{array}\}.$$

The $K_{j(r)}$ factors were calculated on the basis of the above Formula (13), assuming for *σ_φ_*_2_ and *τ_rφ_*_2_, respectively, the values of hoop and tangential stresses obtained with the use of FEM at *n* + 1 nodes.

The accuracy of the determined GSIFs values depends on the selection of the area from which the stresses obtained using FEM are implemented into the approximating functions. This area was determined using the criterion of selecting nodes proposed in [30].

The $K_{j(r)}$ coefficients determined in all selected nodes should theoretically be identical. However, due to potential errors in numerical calculations, the found values of the generalised stress intensity factors may differ slightly. To minimise such an error, the obtained results were averaged according to the following formula:

(14) $$K_{I}=\frac{\sum_{n=1}^{n+1}K_{I(r)}}{n+1},K_{II}=\frac{\sum_{n=1}^{n+1}K_{II(r)}}{n+1}.$$

To verify the method used, the GSIFs were calculated for the problem of a crack perpendicular to the interface and started on it. The obtained results were compared with the exact solution [16] and the approximate one [18]. A satisfactory agreement was obtained in both cases—the difference was about 1.2%. A similar convergence of results was obtained in the notch problem, the tip of which is located on the border of the connection of two materials. The relative difference between the compared GSIFs values, found with the use of the developed method and in the work [26], was about 1.8%.

### 2.3. FEM Modelling

Currently, various types of numerical simulations are often performed before the implementation and production of the product. Numerical calculations can be performed using various methods. One of them is FEM. This method can be used, for example, to analyse issues related to friction [31,32], flow [33,34] or to predict the operation of piezoelectric transducers [35,36,37]. 

**Figure 3:**
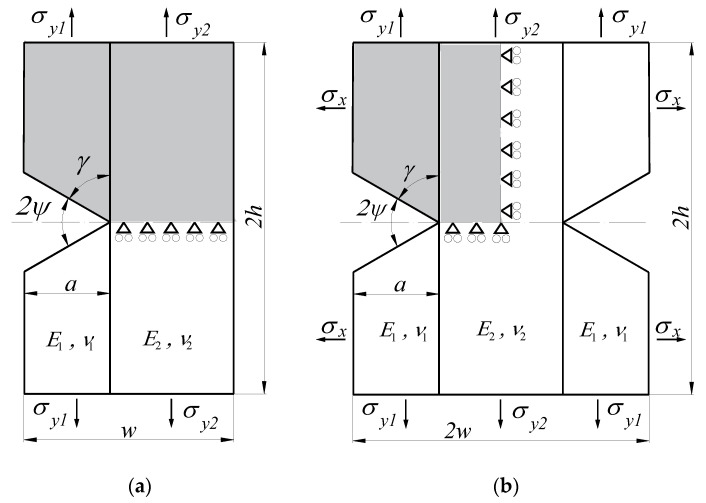
Geometry and method of fixing and loading specimens: (**a**) with a single edge-notched plate, and (**b**) with a double edge-notched plate.

**Figure 4:**
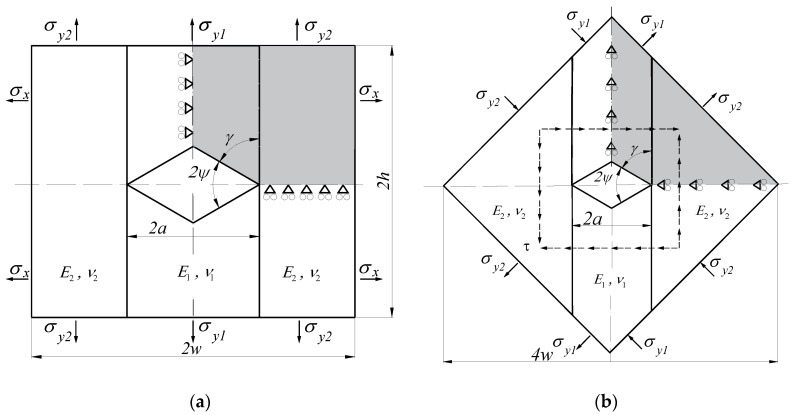
Geometry method of fixing and loading of specimens with a centre-notched plate subjected to (**a**) uniaxial/biaxial tension and (**b**) pure shear loading.

In the presented work, FEM was used to determine generalised stress intensity factors. Numerical calculations were performed in the ANSYS environment, for which, using the built-in programming language Ansys Parametric Design Language (APDL), a proprietary module enabling direct determination of GSIFs was implemented. This module also takes into account the previously mentioned criterion of selecting nodes. The following types of specimens were modelled in numerical simulations:

- a rectangular plate with a single edge sharp corner under uniaxial tension (Figure 3a);
- a rectangular plate with a double edge sharp corner under uniaxial/biaxial tension (Figure 3b);
- a rectangular plate with a central sharp corner under uniaxial/biaxial tension (Figure 4a);
- a rectangular plate with a central sharp corner under pure shear loading (Figure 4b).

As the tested samples have one or two planes of symmetry, only their halves or quarters were modelled (shaded area in the figures above). Symmetry (for tension samples—Figure 3 and Figure 4a) and anti-symmetry (for shear samples—Figure 4b) boundary conditions were assumed in the symmetry planes. 

**Figure 5:**
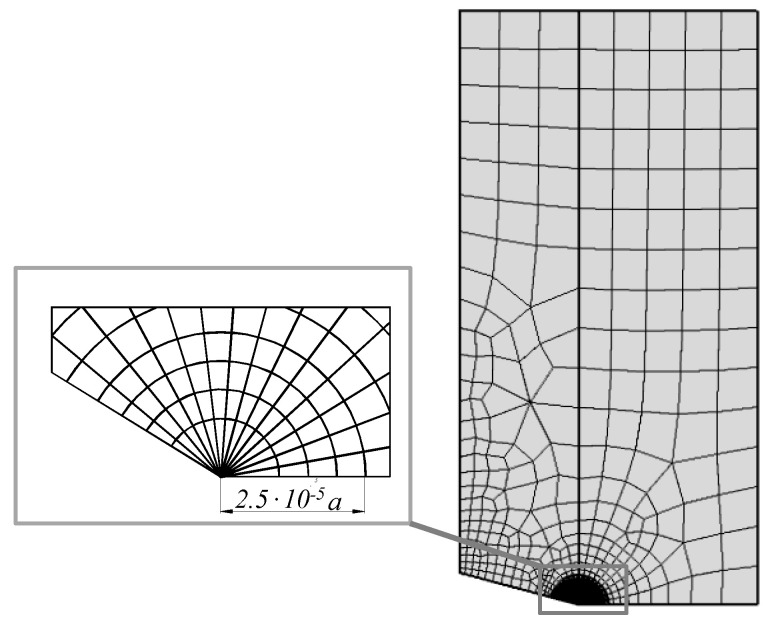
A typical finite element mesh used for modelling the specimens.

The samples were discretized using quadrangular, eight-node finite elements (Figure 5). The mesh of division into finite elements was densified in the tip region. Furthermore, the tip of the notch was surrounded by a special triangular finite element with a shape function, which could simulate the singularities of displacements of the *r*^0.5^ type. In this way, a better representation of the singular stress fields occurring in the vicinity of the corner tip was obtained.

The applied load *σ_y_*_1_ was constant and equal to 1 Pa. However, the value of the load *σ_y_*_2_ was determined on the basis of Formula (15), resulting from the condition of continuity of strain −*ε_y_*_1_ = *ε_y_*_2_:

(15) $$\sigma_{y2}=\frac{E_{2}\left(\sigma_{y1}-\sigma_{x}\nu_{1}\right)}{E_{1}}+\sigma_{x}\nu_{2}.\;$$

For tensile specimens, numerical calculations were performed for variable proportions of longitudinal and transverse loads −*σ_x_*/*σ_y_*_1_. Moreover, the simulations were prepared for various relative stiffness of the individual components of the bi-material–$\Gamma =\mu_{1}/\mu_{2\;},$ where $\mu_{i}=\frac{E_{i}}{2\left(1+\nu_{i}\right)}.$ In all simulations, it was assumed that the Young’s modulus of material 1 was constant and be equal to: *E*_1_ = 1 × 10^9^ Pa. However, the Young’s modulus of material 2 –*E*_2_- was variable and depended on the parameter *Γ*. The Poisson coefficients also depended on this parameter, which were respectively: *ν*_1_ = *ν*_2_ = 0.3 (for *Γ* = 1), *ν*_1_ = 0.3, *ν*_2_ = 0.35 (for *Γ* > 1), and *ν*_1_ = 0.35, *ν*_2_ = 0.3 (for *Γ* < 1).

Numerical tests were performed for specimens with various notch angles *ψ* and proportions of characteristic dimensions *a*/*w*. Moreover, it was arbitrarily assumed that the notch height *a* is constant and amounts to 1 m. The ratio of the height and width of the specimens was also kept constant—*h/w* = 2. The plane stress condition was assumed in all simulations.

## 3. Results and Discussion

The quantitative description of singular stress fields is obtained by finding generalised stress intensity factors. For their determination, using the previously described method, the numerical data (hoop and tangential stresses at the interface of the bi-material) and eigenvalues *λ_j_* are necessary. The latter, for the samples described in Section 2.2, were calculated on the basis of the dependence (7) and (8) and are presented in Table 1 and Table 2.

GSIFs units—[Pa m^1−*λj*^]—depend on the geometrical and material parameters of the specimens. This prevents direct comparison of the results obtained. Such inconvenience can be eliminated by normalising GSIFs [20]. In the presented work, the determined values of the *K_j_* coefficients were normalised using the following formula:

(16) $$F_{j}=K_{j}/\left(\sigma_{y1}\sqrt{\pi }a^{1-\lambda_{j}}\right),\;(j=I,II)$$

The results obtained for each type of specimens are presented below.

### 3.1. Rectangular Plate with a Single Edge Sharp Corner under Uniaxial Tension

Table 3 shows the normalised *F_I_* factors calculated for a tensile bi–material with a single edge notch. The calculations were made for various relative stiffness and notch apex angles. 

By analysing the results presented in Table 3, it can be seen that the normalised *F_I_* values increase with

- an increase in the notch angle 2*ψ*;
- a decrease in the relative stiffness *Γ* (this tendency is consistent with the distribution of the normalised stress intensity factors determined for the crack initiated at the interface [16,18]).

**Figure 6:**
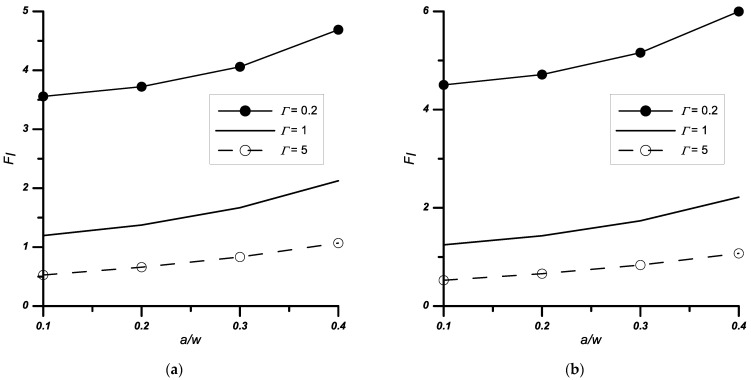
Variation of the normalised stress intensity factors *F_I_* with the relative stiffness *Γ* and notch height *a*/*w*, (**a**) notched bi-material with apex angle 2*ψ* = 30°, (**b**) notched bi-material with apex angle 2*ψ* = 60°.

It was also found that as the height of the notch increased, regardless of the material parameters and its opening angle, the *F_I_* coefficients increased (Figure 6).

### 3.2. Rectangular Plate with a Double Edge Sharp Corner under Uniaxial/Biaxial Tension 

**Figure 7:**
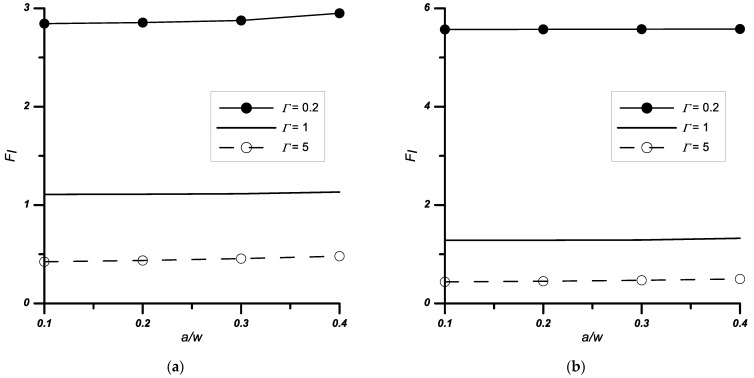
Variation of the normalised stress intensity factors *F_I_* with the relative stiffness *Γ* and notch height *a*/*w*, (**a**) notched bi-material with 2*ψ* = 0° apex angle, (**b**) notched bi-material with 2*ψ* = 90° apex angle.

**Figure 8:**
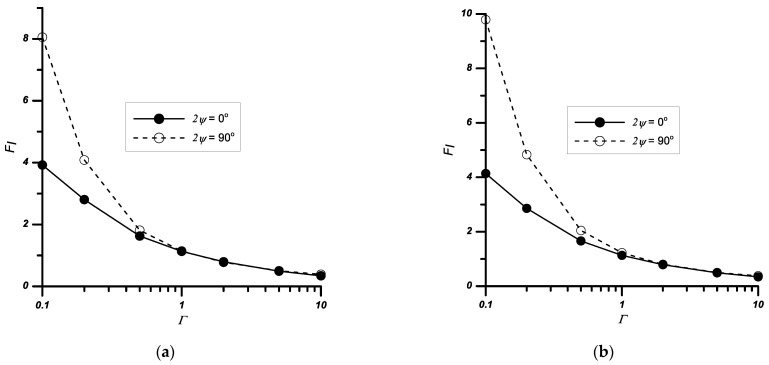
Variation of the normalised stress intensity factors *F_I_* with the relative stiffness *Γ* and the notch angle 2*ψ*, (**a**) *σ_x_*/*σ_y_*_1_ = 1, (**b**) *σ_x_*/*σ_y_*_1_ = 0.5, *a*/*w* = 0.4.

Similar tests, as for the case described in Section 3.1, were performed for the problem of a double edge-notched plate. The obtained results are presented in Table 4 and Figure 7 and Figure 8.

The stress intensity coefficients were calculated for two load variants: uniaxial (*σ_x_* = 0) and biaxial (*σ_x_*/*σ_y_*_1_ ≠ 0) tension. The influence of the tested parameters—relative stiffness *Γ*, vertex angle 2*ψ*, and relative notch height *a/w*—on the values of normalised stress intensity factors was identical as for the single edge-notched plate problem. 

As for the influence of the ratio of loads perpendicular and parallel to the interface (*σ_x_*/*σ_y_*_1_) on the values of the *F_I_* coefficients, on the basis of the analyses performed (Table 4, Figure 8) it can be concluded that with increasing load *σ_x_*:

- the *F_I_* value significantly decreases for *Γ* ≤ 1;
- increases slightly for the case where *Γ >* 1.

Of course, for the case of a crack in a homogeneous material (*Γ* = 1, 2*ψ* = 0°), the application of an additional load *σ_x_* does not affect the stress intensity factor.

### 3.3. Rectangular Plate with a Central Sharp Corner under Uniaxial/Biaxial Tension 

**Figure 9:**
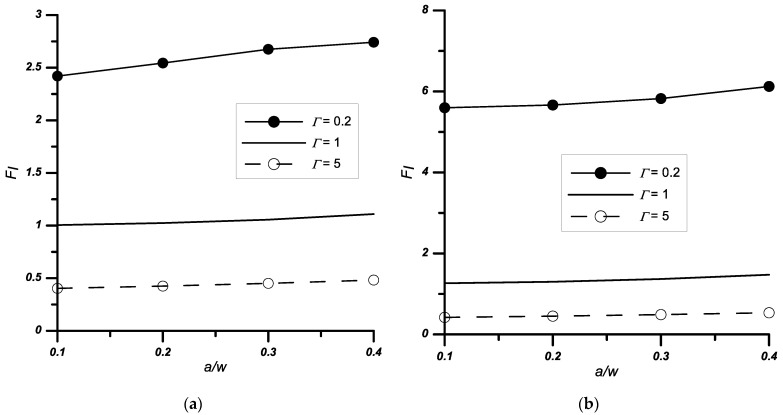
Variation of the normalised stress intensity factors *F_I_* with the relative stiffness *Γ* and notch height *a*/*w*, (**a**) notched bi-material with apex angle 2*ψ* = 30°, (**b**) notched bi-material with apex angle 2*ψ* = 90°.

**Figure 10:**
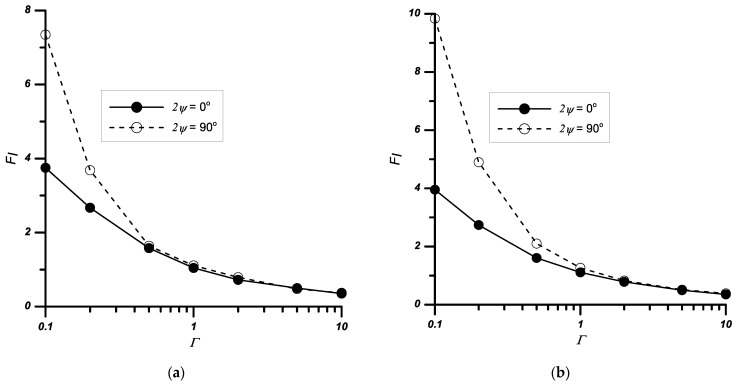
Variation of the normalised stress intensity factors *F_I_* with the relative stiffness *Γ* and the notch angle 2*ψ*, (**a**) for *σ_x_*/*σ_y_*_1_ = 1, (**b**) for *σ_x_*/*σ_y_*_1_ = 0.5, *a*/*w* = 0.4.

The same tests, as for the case described in Section 3.2, were performed for the problem with the centre-notched plate. The obtained results are presented in Table 5 and Figure 9 and Figure 10. 

The performed analyses showed that the factors *F_j_* always increase with a decrease in the relative stiffness *Γ* and an increase in the height of the notch and its apex angle.

For samples subjected to the biaxial tension load, similarly to the problem of the double-sided notch, an increase in the load *σ_x_* causes either a decrease in the values of the *F_I_* (for *Γ* ≤ 1) coefficients or their increase (for *Γ* > 1).

### 3.4. Rectangular Plate with a Central Sharp Corner under Pure Shear Loading 

**Figure 11:**
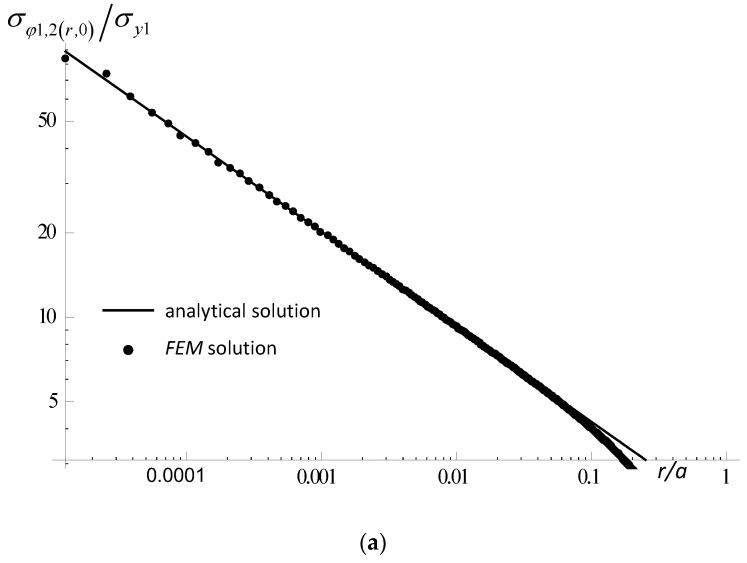

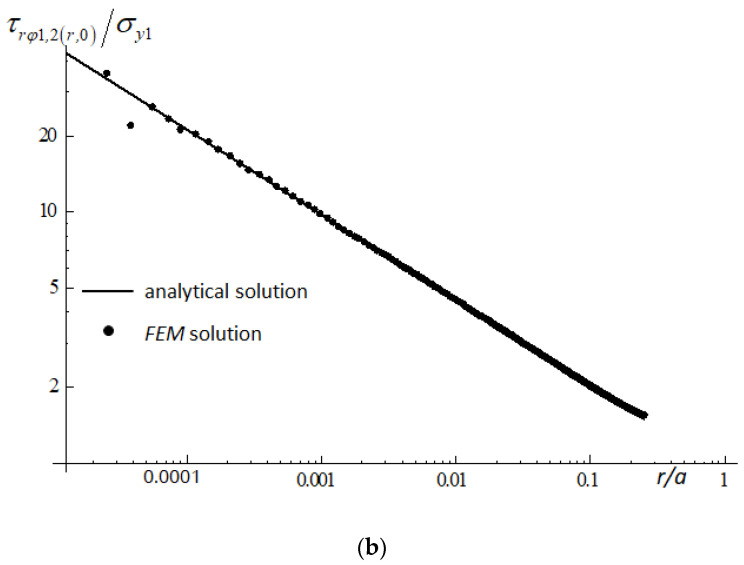
Comparison of selected stress distributions (pure shear loading) obtained by the FEM (points) and according to the analytical Formula (11) using the factors from Table 6, (continuous lines), (**a**) hoop stress, (**b**) tangential stress, (*φ* = 0°, 2*ψ* = 30*°*, *Γ* = 0.5).

The test results for the notched samples subjected to tangential load (Mode II) are shown in Table 6 and Figure 11.

As for elements subjected to shear loads, the values of the normalised factors *F_II_* (Table 6) change in the same way as in the case of applying tensile loads (increase with the decrease of the relative stiffness and the increase of the notch tip angle).

In Figure 11, for an element with a central notch subjected to pure shear (for arbitrarily selected geometric and material parameters), the stress distributions obtained from the analytical solution were compared with the results obtained through FEM. Good agreement of both solutions was obtained in the apex region of about 10% of the notch height. Similar comparisons were also made for the specimens discussed in Section 3.1, Section 3.2 and Section 3.3 (not included in the paper). In each case, a similar compliance of the analytical description with the FEM solution was obtained.

## 4. Conclusions

The paper presents analytical and numerical solutions for the plane problem of a sharp corner, assuming that its tip touches the line separating two different elastic materials. 

Two variants of loading were considered—tensile (transverse and/or longitudinal to the interface) and shear in the plane of symmetry of the notch. For both load cases, eigenequations were determined, which depended on the mechanical properties of both components of the composite and the notch tip angle. It was found that for each loading variant, there is always one singular term described by the real eigenvalues *λ_j_*. Moreover, it was observed that the stress singularity strength decreased with increasing notch apex angle and relative stiffness *Γ*.

Furthermore, formulas were derived to determine the individual components of the stress tensor occurring in the notch tip area with the use of generalised stress intensity factors *K_j_*. The *K_j_* coefficients were also calculated for three variants of the notch location: a single edge sharp notch, a double edge sharp, and a central sharp notch. The calculations were made for various notch apex angles as a function of material constants. 

It was found that the normalised values of generalised stress intensity factors increase with a decrease in the relative stiffness *Γ* and an increase in the notch height and its apex angle. Moreover, it was found that in the case of specimens subjected to biaxial tension, an increase in the load perpendicular to the interface (*σ_x_*) causes either a decrease in the values of the normalised stress intensity factors (*Γ* ≤ 1) or their increase (*Γ* ≤ 1).

The derived analytical solution describing the individual stress components was compared with the results obtained using the FEM. Both solutions were found to be very compatible in the apex region of about 10% of the notch height. 

The research results presented in this article can be used by other researchers in many ways, e.g., as comparative data. The analytical and numerical description of singular stress fields can also be used to develop a fracture criterion of structural elements with this type of material defect. The development of such a criterion will be the aim of the future works of the author. 

## Figures and Tables

**Table 1:** The eigenvalues *λ_I_* (Mode I).

2*ψ* [°]	*γ* [°]	*λ_I_*						
		*Γ* = 0.1	*Γ* = 0.2	*Γ* = 0.5	*Γ* = 1	*Γ* = 2	*Γ* = 5	*Γ* = 10
0	90	0.68145	0.64075	0.56383/0.56383 *	0.5	0.42944/0.42944 *	0.32579	0.25150
30	75	0.72936	0.67111	0.57386	0.50145	0.43042	0.33514	0.26647
60	60	0.79536	0.72026	0.59975	0.51222	0.43166	0.33627	0.27426
90	45	0.86612	0.78694	0.64984	0.54448	0.44639	0.33606	0.27385

*ν*_1_ = 0.3, *ν*_2_ = 0.35 for *Γ* > 1, *ν*_1_ = 0.35, *ν*_2_ = 0.3 for *Γ* < 1; *—from reference [18].

**Table 2:** The eigenvalues *λ_II_* (Mode II).

2*ψ* [°]	*γ* [°]	*λ_II_*						
		*Γ* = 0.1	*Γ* = 0.2	*Γ* = 0.5	*Γ* = 1	*Γ* = 2	*Γ* = 5	*Γ* = 10
0	90	0.68145	0.64075	0.56383	0.5	0.42944	0.32579	0.25150
30	75	0.77821	0.73773	0.66074	0.59819	0.52828	0.41805	0.33128
60	60	0.92776	0.88173	0.79616	0.73090	0.66201	0.55226	0.45604
90	45	1	1	0.99105	0.90853	0.83206	0.73052	0.64428

*ν*_1_ = 0.3, *ν*_2_ = 0.35 for *Γ* > 1, *ν*_1_ = 0.35, *ν*_2_ = 0.3 for *Γ* < 1.

**Table 3:** Values of normalised stress intensity factors *F_I_* calculated for a rectangular plate with a single edge sharp corner under uniaxial tension, *a*/*w* = 0.2.

Γ	*F_I_*			
	2*ψ* = 0°	2*ψ* = 30°	2*ψ* = 60°	2*ψ* = 90°
0.1	4.317	6.070	8.765	11.990
0.2	3.076	3.722	4.713	5.991
0.5	1.908	2.019	2.236	2.609
1	1.361	1.375	1.431/2.220 **	1.579/2.471 **
	1.367 *		2.230 *	2.478 *
2	0.983	0.983	0.989	1.042
5	0.636	0.659	0.659	0.659
10	0.452	0.490	0.502	0.504

*—From reference [38]; **—calculated for *a/w* = 0.4.

**Table 4:** Values of normalised stress intensity factors *F_I_* calculated for the rectangular plate with a double edge sharp corner under uniaxial/biaxial tension, *a*/*w* = 0.4.

*Γ*	*F_I_*							
	2*ψ* = 0°		2*ψ* = 30°		2*ψ* = 60°		2*ψ* = 90°	
	*σ_x_* = 0	*σ_x_*/σ_y__1_ = 2	*σ_x_* = 0	*σ_x_*/*σ_y_*_1_ = 2	*σ_x_* = 0	*σ_x_*/*σ_y_*_1_ = 2	*σ_x_* = 0	*σ_x_*/*σ_y_*_1_ = 2
0.1	4.332	3.488	5.847	3.68	8.469	4.086	11.498	4.600
0.2	2.950	2.430	3.489	3.00	4.412	2.424	5.580	2.578
0.5	1.685	1.525	1.767	1.40	1.958	1.318	2.281	1.343
1	1.132	1.132	1.140	1.03	1.189	0.950	1.324	0.969
2	0.772	0.806	0.777	0.778	0.781	0.783	0.821	0.832
5	0.480	0.493	0.497	0.51	0.496	0.509	0.517	0.518
10	0.344	0.346	0.368	0.38	0.376	0.395	0.381	0.406

**Table 5:** Values of normalised stress intensity factors *F_I_* calculated for the rectangular plate with a central sharp corner under uniaxial/biaxial tension, *a*/*w* = 0.4.

*Γ*	*F_I_*							
	2*ψ* = 0°		2*ψ* = 30°		2*ψ* = 60°		2*ψ* = 90°	
	*σ_x_* = 0	*σ_x_*/*σ*_*y*1_ = 2	*σ_x_* = 0	*σ_x_*/*σ*_*y*1_ = 2	*σ_x_* = 0	*σ_x_*/*σ*_*y*1_ = 2	*σ_x_* = 0	*σ_x_*/*σ*_*y*1_ = 2
0.1	4.146	3.395	5.818	3.766	8.740	3.793	12.36	2.415
0.2	2.742	2.410	3.497	2.460	4.600	2.300	6.12	1.270
0.5	1.620	1.500	1.767	1.464	2.067	1.304	2.56	0.710
1	1.109 1.004 *	1.109	1.146 1.027 *	1.076	1.254 1.112 *	0.953	1.47 1.263 *	0.596
	0.996 **		1.028 **		1.115 **		1.267 **	
2	0.771	0.803	0.782	0.797	0.818	0.821	0.91	0.512
5	0.482	0.505	0.498	0.526	0.502	0.508	0.53	0.406
10	0.345	0.353	0.366	0.381	0.379	0.385	0.39	0.337

**—From reference [39], *—calculated for *a/w* = 0.1.

**Table 6:** Values of normalised stress intensity factors *F_II_* calculated for a rectangular plate with a central sharp corner under pure shear loading, *a*/*w* = 0.4.

*Γ*	*F_II_*			
	2*ψ* = 0°	2*ψ* = 30°	2*ψ* = 60°	2*ψ* = 90°
0.1	13.739	16.146	19.152	-
0.2	6.501	7.796	9.387	-
0.5	2.279	2.845	3.530	4.669
1	1.034	1.345	1.731	2.124
2	0.451	0.613	0.836	1.074
5	0.139	0.197	0.293	0.425
10	0.062	0.088	0.135	0.218

## Data Availability

The data presented in this study are available on request from the corresponding author. At the time the project was carried out, there was no obligation to make the data publicly available.
